# Author Index for Volume 89

**DOI:** 10.1038/sj.bjc.6601554

**Published:** 2003-12-09

Abbady, A-Q 120

Abbas, MT 1638

Abbruzzese, JL 8

Abe, N 2104

Abe, S 1008

Abe, T 1172

Abecassis, J 1940

Abken, H 1130

Aboagye, EO 1327

Abou El Hassan, MAI 357, 2140

Açil, Y 1722

Adab, F 1155

Adami, H-O 1221

Adami, HO 775

Adami, J 1221

Adams, J 88

Addington-Hall, JM 2190

Agerbaek, M 298

Aguado, T 101

Aguggini, S 977

Aguirre-Cruz, L 2327

Åhlén, J 720

Ahlman, H 460, 1383, 2093

Ahmed, N 374

Ahmed, S 2197

Ahmed, SB 1502

Aillères, N 1987

Airley, R 870

Aitchison, M 1906

Aitken, M 2197

Ajisaka, H 676

Akazawa, K 546

Akre, O 1664

Akslen, LA 1031

Alaiya, AA 305

Alderson, D 308, 497

Alfaro, M 1248

Allardice, GM 505

Allevi, G 977

Allum, W 1035

Almog, B 109

AL Moneef, M 2271

Alquati, P 977

Alsner, J 298

Altieri, DC 2244

Altiparmak, G 2093

Altman, DG 232, 431, 605, 781

Altura, RA 1743

Alves, P 199

Amariglio, N 314

Amat, S 1185

Ambrogi, F 268

Amir, Z 15

Anagnostaki, L 1920

Anderson, E 653, 1650

Anderson, H 262, 1155, 2190

Andersson, J 460

Angerson, WJ 1028

Anné, J 1796

Ansell, P 1228

Anton-Culver, H 1513

Antonelli, A 1091

Antonuzzo, A 477

Aparicio, T 1439

Appleton, S 653, 1400

Aranes, MJ 1757

Aránega, A 192

Arango, D 1757

Archer, CD 1035

Areces, L 930

Arich, A 1552

Arita, K 1802

Arlt, A 1714

Armbruster, C 702

Armstrong, BK 1450

Artru, P 1439

Ashley, S 1035

Ashman, JNE 864

Ashton, T 1314

Astrup, L 252

Ateenyi-Agaba, C 423

Atkins, GJ 206

Atkins, H 2312

Atomi, Y 2104

Attems, J 983

Attisano, L 1538

Atzpodien, J 50

Aubert, G 577

Auer, G 720

Augenlicht, LH 1757

Augustsson, K 775

Auperin, A 1102

Autio, V 1966

Avivi, I 1389

Avril, A 666

Aytac, U 1366

Azaiez, R 1502

Azizuddin, K 1590

Azria, D 1987

Azuma, K 1079

Baba, S 730

Bach Hansen, J 252

Backman, H 971

Bai, J 2227

Bakker, PJM 2219

Balkwill, F 1418

Balogh, á 465

Balzarotti, M 1159

Banura, C 65

Bar-Am, A 109

Barbera, S 1013

Bar-Chana, M 1657

Barr, H 106

Barrett-Lee, P 1834

Barthel, B 564

Barthel, H 1327

Bartlett, JMS 552, 1305

Bartolini, S 246

Barua, AB 412

Bar-Yehuda, S 1552

Basnett, I 492

Bast, A 357, 2140

Bastian, J 405

Bates, SE 1971

Batlle, A 173

Battermann, JJ 2184

Batty, GD 1243

Bauer, J 1620

Baxevanis, CN 1055

Baxter, JN 1314, 1729

Beck, J 1855

Becker, JC 2019

Becker, W 2234

Beckett, L 2110

Bedard, P 641

Beer, TM 968

Beex, LVAM 271

Begent, RHJ 1849

Bégin, LR 1031

Beijnen, JH 1776

Beinke, C 2122

Bekkers, RLM 886

Belembaogo, E 1185

Bell, CMJ 492

Bell, J 508

Bella, SD 1463

Belldegrun, A 1598

Belloc, J 1439

Bellocco, R 1697

Bénard, J 1108

Benghiat, A 70

Benjamin, E 2249

Bennett, MW 1345

Beral, V 423, 502, 2078

Beretta, GD 1428

Berger, R 314

Bergeron, C 470, 1605

Berghmans, T 55, 959

Bergman, R 1657

Berman, ML 1062

Bermudez Moretti, M 173

Bernard-Gallon, DJ 168

Bernengo, MG 1457

Berney, D 1140

Bernhard, H 630

Bernhardt, P 1383

Berns, EMJJ 2289

Bernstein, JL 1513

Bernstein, L 1513

Berrington de Gonzalez, A 519, 2078

Berruti, A 977

Bersiga, A 977

Bertoli, G 977

Bertuzzi, A 1159

Bessho, A 795

Biasco, G 477

Bicknell, R 228, 1927

Biganzoli, E 268

Bignon, Y-J 168

Biondi, A 763

Birch, JM 603

Bird, K 1048

Biroccio, A 1091

Bishop, L 36

Biswas, S 455

Bjørge, T 1238

Blackhall, FH 1152

Blagden, SP 1022

Blagosklonny, MV 1147

Blazeby, JM 497

Bleecker, ER 1524

Blijham, GH 2234

Body, J-J 178

Boehlke, I 29

Boezen, HM 1192

Boffetta, P 1209

Boice, Jr, JD 1513

Bokemeyer, C 29, 1141, 2051, 2133

Boku, N 2207

Boland, GP 277

Bold, RJ 391, 2110

Bolsi, G 977

Bomsztyk, K 1493

Bonardi, S 977

Bond, E 1855

Bonneton, C 539

Boracchi, P 268

Borle, F 2320

Bornstein, J 109

Børresen-Dale, A-L 1513

Borrill, C 15

Bosch, X 1667

Boshoff, C 423, 502, 2284

Botha, JL 70

Bottini, A 977

Bouaouina, N 1502

Bouchardy, C 2023

Bouchier-Hayes, L 1327

Boué, DR 1743

Bougioukas, G 877

Boulaiz, H 192

Boulay, J-L 422

Bouralexis, S 206

Bourboulia, D 423, 502

Bourhis, J 1102

Bousarghin, L 423

Bower, M 457

Bowman, KJ 2271

Boxall, J 1901

Boyd, J 505,

Boyd, NF 1672

Boyle, P 2087

Bozzino, J 1155

Brabender, J 1508

Bracco, L 1940

Bradburn, MJ 232, 431, 605, 781

Bradbury, I 224

Bradley, C 1155

Brady, F 1327

Braga, C 94

Brain, K 1834

Brand, H 1130

Brandi, G 477

Branle, F 55

Brännström, M 1298

Bratti, MC 1248

Braun, MS 1155

Bravou, V 2341

Braybrooke, JP 1166

Breda, E 1180

Bredenkamp, R 630

Breen, M 1530

Brennan, P 1209

Brenner, H 1260

Brewer, C 1062

Brewster, DH 505

Bria, E 1870

Brickell, K 615

Bridger, JE 113

Brismar, K 1697

Brizzi, MP 977

Bröcker, EB 2019

Broggini, M 763

Bromwich, EJ 1906

Bronner, C 120

Brookes, ST 497

Brosjö, O 720

Browaeys, P 727

Brown, JE 2031

Brown, M 1906

Brown, RJ 524

Brown, SB 398

Brunet, J-S 1031

Brurberg, KG 350

Bruyns, C 727

Buckle, T 1776

Budman, DR 1566

Bugat, R 997

Bui, BN 2213

Bulk, S 834

Bulten, J 886

Bümming, P 460

Bunce, M 1096

Bunch, KJ 1215

Bundgaard, H 1633

Bundred, NJ 277

Burcharth, F 252

Burchill, SA 1276

Burger, AM 1538

Burk, RD 1248

Burke, B 421

Burke, P 1645

Burnett, RD 808

Burrell, HC 1310

Burtles, SS 437

Bussink, J 271

Bussolati, G 930

Buthaud, X 2057

Butow, P 2069

Butow, PN 1450

Buzzi, F 992

Calandri, C 246

Callaghan, R 1581

Calvanese, A 239

Cameron, DA 1837

Campbell, E 222, 573

Campbell, H 653, 1400, 1650

Campbell, J 653

Campbell, L 1909

Campbell, NC 821

Canna, K 612, 1906

Cany, L 2213

Cao, Y-D 2227

Capdeville, R 1855

Cappuzzo, F 246

Carlsson, P 1298

Caroli, UM 1620

Carpenter, L 423, 502

Carrillo, E 192

Carvalho, G 1108

Casabonne, D 423, 502

Casado, A 1002

Casado, MA 1002

Casals, J 2023

Casas, A 173

Cascinu, S 1428

Cassoni, P 930

Castagna, L 1159

Castaldo, V 1827

Castellsagué, X 1209, 1667

Castiglione, F 1013

Castle, PE 1248

Catalano, V 1428

Catane, R 997

Catania, C 1403

Catovsky, D 36

Catt, S 1400

Cawkwell, L 864

Ceciarini, F 239

Cella, D 641

Celli, N 763

Cerretani, D 239

Cetnarskyj, R 653, 1650

Chae, K-Y 907

Chalabi, N 168

Chan, JK 1062

Chan, KC 277

Chan, S 660

Chan, SY 284

Chang, BL 1524

Chang, JT 681

Chang, YF 1995

Chang, Y-L 320

Chao, P-C 146

Chappuis, PO 1031

Charman, SC 1022

Charpentier, B 1108

Chastagner, P 470, 1605

Chatrath, P 1048

Chau, I 36

Chauveinc, L 2057

Checkley, D 1889

Chelkowska, E 777

Chen, I-H 681

Chen, S 1248

Chen, W-F 291

Chen, WY 847

Chen, W-Z 2227

Chen, X 617

Chen, Z 1865

Cheng, A-J 681

Cherbonnier, C 1108

Chern, YT 1995

Cheung, ANY 697

Cheung, KL 284, 660

Chi, CW 1995

Chiappetta, G 2104

Chilvers, C 2078

Chini, B 930

Chinol, M 930

Chiu, PM 697

Chiu, S-M 1590

Cho, Y 1736

Cho, YG 1958

Chollet, P 1185

Chou, J-M 146

Chouchane, L 1502

Christensen, IJ 420

Christgen, M 1714

Christian, J 258

Christodoutous, K 1096

Chye, R 2069

Ciatto, S 1645

Ciccolini, J 585

Cigolari, S 1013

Clamp, AR 1152

Clark, IM 1812

Clark, PI 43

Clark, TG 232, 431, 605, 781

Clayman, B 1248

Clayton, J 2069

Clemens, M 997

Clerici, M 1463

Clifford, GM 101

Clifton, L 455

Cnattingius, S 1664

Coindre, JM 666

Colditz, GA 847

Coleman, N 1048

Coleman, R 1152

Coleman, RE 2031, 2197

Collingridge, DR 1327

Collins, S 77

Comella, P 992

Communal, Y 168

Compasso, S 1159

Concannon, P 1513

Conde, M 1667

Condon, LT 864

Connault, E 1102

Connelly, BS 1672

Cook, G 258

Cooper, R 870

Coradini, D 268

Corbex, M 1502

Cordes, N 2122

Cordon-Cardo, C 2172

Corner, GA 1757

Cornford, EJ 1310

Correa García, S 173

Correa, P 1209

Correale, P 199, 239

Corrie, P 1418

Corrie, PG 455

Cotter, TG 1950

Couette, JE 1638

Counsell, R 1155

Coursaget, P 423

Coventry, BJ 533

Cox, S 457

Coze, C 1605

Cramer, SD 746

Cranston, D 1096

Cree, IA 2299

Crépin, M 215

Crinière, E 2327

Crinò, L 246

Crocetti, E 94

Cromer, A 1940

Crossley, B 2078

Crow, JC 2249

Crow, P 106

Cruickshank, ME 966

Cull, A 653, 1400

Cunningham, D 36

Curé, H 1185

Curiel, DT 1162

Curigliano, G 1403

Currie, EJ 1423

Curtin, JF 1950

Cusi, MG 199

Cuzick, J 88

Czerwenka, K 983

Dabrosin, C 385

D'Agostino, G 1180

Dahm-Daphi, J 593

Daidone, MG 268

Daley, FM 1290

Dalhoff, K 252

Daling, JR 513

Dal Maso, L 94

Damato, BE 1961

Damera, A 1310

Dancey, J 252

Danenberg, KD 1508

Danenberg, PV 1508

Danesi, R 1403

Dang, NH 1366

Daugaard, G 1633

Dauplat, J 1185

Davey, HM 1450

Davey, Smith, G 81, 1243

Davies, BR 2312

Davies, RJ 1048

Davies, S 1155

Davies, T 1693

Davis, AJ 617

Davis, DW 8

Davis, JM 2069

Dawson, J 15

Day, K 891

Day, N 1228

Day, NE 1

Déas, O 1108

de Braud, F 1403

de Bruijn, E 1796

de Graeff, A 2219

de Gruijl, TD 1162

de Haes, JCJM 2219

de Jong, S 363

De la Rochefordière, A 2057

de Latour, M 1185

De Lucia, L 992

De Maio, E 1013

de Mascarel, I 666

De Pas, T 1403

De Pooter, CMJ 2305

de Sande, LM 1002

De Stavola, BL 508, 852

De Stefani, E 1209

de Valk, B 243

de Vathaire, F 840, 1638

De Vita, F 992

de Vries, EGE 363, 1192

Deacon, J 2078

Debatin, K-M 2155

Debiec-Rychter, M 1409

Debled, M 2213

Debrock, G 1409

Defachelles, AS 1605

Delaney, E 966

Delattre, J-Y 2327

Dellatolas, G 2038

Delmotte, P 959

Delvenne, P 899

Deneo-Pellegrini, H 1209

Denton, G 1130

Deo, YM 2234

Deplanque, G 1166

Desramé, J 1439

Deutsch, E 1102

Dhillon, T 779

Di Betta, D 997

Di Maio, M 1013

Di Marco, MC 477

Dickman, PW 775

Dickson, RB 1479

Dicterow, E 1473

Didilis, V 877

Dikomey, E 593

Dilhuydy, JM 666

D'Incalci, M 763

Di Nicolantonio, F 2299

Dinjens, WNM 2289

Di Palma, T 239

Dite, GS 1661

Doak, SH 1729

Dogliotti, L 977

Dominguez, S 1439

Donaldson, A 308

Dondon, MG 1638

Dong, X-Y 291

dos Santos Silva, I 508, 852

Dottorini, ME 1638

Dowsett, M 1035

Draper, GJ 1215

Dresselaers, T 1796

Dreznick, Z 1552

Driessens, G 727

Dryver, ET 482

Dubois, JB 1987

Dubreuil, A 585

Ducreux, M 997

Duff, SE 426

Duffy, SW 1693

Dunckley, M, 1400

Dunlap, N 746

Dunlop, DJ 1028

Dunlop, M 1400

Dunn, I 1305

Dunne, AL 2277

Dunsche, A 1722

Duprez, A 2327

Durand, M 666

Dürrbach, A 1108

Dutreix, C 1855

Dutrillaux, AM 2327

Dwek, MV 305

Dwyer, J 1255

Eads, D 746

Earl, H 1418

Easton, DF 1961

Ebara, M 1086

Ebbs, SR 1035

Ebrahim, S 81

Eccles, D 308

Eccles, M 2163

Echner, H 1055

Eden, OB 1228

Edwards, DR 1812

Edwards, J 552

Eggert, AO 2019

Eguchi, K 795

Egyházi, S 1517

Eigentler, TK 1620

Eilers, KM 968

Eisenbach, L 314

Ekbom, A 1221, 1664

Ekström, K 1221

El-Abbadi, MM 1375

El-Awady, RA 593

Elder, DJE 1358

Elli, R 1091

Ellis, IO 1310

Ellis, PA 1035

Ellis, SP 2031

El Sharouni, SY 2184

Elstrodt, F 2289

Engeland, A 1238

English, MA 1215

Engström, K 460

Eppinga, P 1192

Erba, E 763

Erdas, R 268

Eremenco, S 641

Erjavec, Z 1192

Erlinger, S 252

Ernst, T 687

Eskens, FALM 2045

Etienne, M-C 585

Etienney, I 1439

Evans, A 891

Evans, AJ 284, 660, 1310

Evans, GS 1123

Evans, SS 1789

Evans, TRJ 252

Evdokiou, A 206

Everard, J 2197

Eves, P 2004

Ezenfis, J 1439

Facey, K 224

Fages, B 997

Fahy, BN 391, 2110

Fainaru, I 808

Fainaru, O 109

Faircloth, GT 763, 2305

Falcini, F 94

Falcone, A 477

Falcou, MC 2057

Fallowfield, L 1445

Fan, Z 185

Farewell, V 1445

Farmer, JAM 106

Farris, A 992

Fayyazi, A 915

Fear, NT 1228

Fedier, A 1559

Fegg, MJ 2202

Feik, E 702

Fernö, M 1920

Ferrandina, G 1180

Ferrari, AC 1566

Ferraris, C 1463

Ferrière, J-P 1185

Ferrière, JM 2213

Feskanich, D 847

Fiegl, H 1934

Fierro, MT 1457

Figer, I 997

Figlin, RA 1598

Findlay, DM 206

Fink, D 1559

Firat, H 199

Fischel, J-L 585

Fischer, DC 1927

Fischer, T 1855

Fishman, P 1552

Flavell, KJ 1200

Floquet, A 666

Flynn, S 2087

Fontolliet, C 2320

Foo, K 1433

Forman, D 487

Formento, J-L 585

Formento, P 585

Forrest, LM 1028

Forssell-Aronsson, E 1383

Fosså, SD 1145

Foster, T 248

Foulkes, WD 1031

Fowst, C 1559

Franc, B 2098,

France, E 1834

Franceschi, S 94, 101, 1667

Francini, G 199, 239

Francoual, M 585

Frants, RR 1914

Frascogna, V 1102

Friedman-Birnbaum, R 1657

Fromigue, O 178

Frontini, L 1013

Fry, A 653, 1650

Fujii, S 2293

Fujimoto, S 1896

Fujise, Y 730

Fujita, A 1008

Fujita, T 546

Fujiyama, S 1614

Fukai, Y 140

Fukuchi, M 140

Fukuhara, K 2293

Fukushima, N 338

Fuller, S 1849

Fusco, A 2104

Fustier, P 168

Füzün, M 870

Gäbel, H 1221

Gabellini, C 1091

Gaffan, J 1849

Gago-Dominguez, M 1686

Gaipa, G 763

Galakhin, K 2098,

Galibert, F 1530

Galli, C 477

Gallo, C 1013

Gambardella, A 992

Gamzu, R 109

Ganesan, TS 808, 1166

Ganser, M 1130

Garambois, V 1987

Garbe, C 1620

García, áM 192

García-Borrón, JC 2004

Garcia-Caballero, T 524

Garden, OJ 1423

Garufi, C 1870

Garzotto, M 968

Gaston, R 2213

Gatter, KC 877

Gatti, RA 1513

Gauldie, J 385

Gazzard, B 457

Gbaguidi, N 1180

Gejyo, F 803

Gemba, K 1885

Generali, D 977

Gennaro, M 1463

Gentaz, E 2038

Geoerger, B 577

Gerhard, MC 2147

Gerl, A 2202

Germano, IM 922

Geroni, C 1559

Gerritsen, WR 577

Gerst, B 1766

Gewies, A 1574

Ghanem, G 2004

Ghisdal, L 55

Giannakopoulos, X 727

Giatromanolaki, A 877

Giles, GG 1661

Gilligan, D 1022

Ginanni, V 1159

Giorgi, G 239

Giovannucci, E 1705

Gips, M 997

Girodet, J 2057

Glare, P 2069

Glaser, M 1327

Glasmacher, A 1413

Glimelius, B 1221

Glück, R 199

Goding, CR 1072

Godward, S 1693

Goebel, G 1934

Goffin, JR 1031

Goggins, M 338

Goldberg, DJ 505

Goldstone, A 1389

Gonzalez, MA 455

Goodall, J 1072

Goodship, A 2284

Gordois, A 634

Gorman, D 653, 1650

Gorschlüter, M 1413

Goto, M 816

Goto, T 1627

Gotoh, M 2207

Graff, BA 350

Gramatzki, M 2234

Granath, F 1221

Grant, JW 1048

Gray, J 1834

Graziano, F 1428

Greco, M 1463

Green, H 1961

Green, J 2078

Greenman, J 864

Gridelli, C 1013, 1827

Griffin, BE 113

Griffiths, AP 1314, 1729

Griffiths, DFR 1909

Grigor, KM 552, 1305

Grill, J 577, 2038

Grimm, S 1574

Görken, IB 870

Gröne, E 687

Gröne, H-J 687

Groen, HJM 1192

Groenewegen, G 2234

Gruber, U 2202

Gruis, NA 1914

Grundy, RG 1200

Gsur, A 702

Guan, Z 1865

Gueders, M 899

Gui, G 1035

Gumbleton, M 1909

Gunnell, D 81

Gunning, PW 860

Günsberg, P 1352

Guo, D 1285

Guo, M 672

Gutcher, SA 2031

Gutteridge, E 284, 660

Guttman-Yassky, E 1657

Guzzinati, S 94

Haan, RJ 2219

Habbema, JDF 1830

Habrand, J-L 2038

Häcker, G 2147

Haeffner, A 1108

Hagmar, L 774

Haidinger, G 702

Haile, RW 1513

Halbert, G 1152

Haldane, JS 113

Haldar, NA 1096

Hall, P 1638

Haller, U 1559

Hama, S 1802

Hamaguchi, T 816

Hamberg, KJ 252

Hamblin, MR 937

Hamel, N 1031

Hamid, AA 2293

Hamma-Kourbali, Y 215

Han, C 1166, 1418

Hancock, B 1418

Hancock, BW 2197

Hanisch, F-G 1130

Hansen, HH 1633

Hansmeier, B 2122

Hansson, J 1517

Harada, M 795

Harada, T 2116

Hardy, R 852

Harguindey, S 1395

Harington, JS 777

Harita, S 795

Harkes, IC 2289

Harmelin, A 314

Harmer, C 258

Harper, SM 1789

Harris, AL 2, 262, 808, 877, 1096, 1166, 1418

Harsan, L 727

Hartmann, JT 29, 2051

Hartmann, M 1405

Hartmann, O 470, 1605

Hasan, T 937

Hasegawa, H 158

Hashida, H 158

Hassan, AB 1860

Hata, K 1232

Hattori, N 158

Haward, R 15

Haward, RA 487

Hawkins, GA 1524

Hay, S 206

Haycock, J 2004

Haylock, RGE 1215

Hearle, N 308, 1961

Hedin, L 1298

Hedman, H 1285

Heerschap, A 754

Heike, Y 1802

Helal, AN 1502

Helenius, H 971

Helin, H 1266

Hellberg, P 1298

Henderson, S 2284

Henner, WD 968

Hennig, M 630

Henriksson, R 1285

Herbst, RS 8

Hergenhahn, M 687

Hermon, C 2078

Herr, I 2155

Herrero, R 1248, 1667

Herrle, F 1927

Herrmann, R 422

Heuser, C 1130

Hewitt, D 2284

Hicks, DJ 1358

Hida, Y 1042, 1736

Hiddemann, W 2202

Hidvégi, M 465

Hihara, J 1876

Hildesheim, A 1248

Hiraga, H 1072

Hirakawa, T 546

Hirakawa, Y 1545

Hiraki, S 795

Hiraki, Y 795

Hiraoka, K 1736

Hirayama, R 1486

Hirsch, F 1108

Hirst, DG 2264, 2277

Hirvikoski, P 1266

Ho, ETS 2264

Hoare, S 1418

Hodgson, SV 308

Hoeben, RC 2140

Hoffmann, A 465

Hofheinz, RD 2051

Hojilla, CV 1817

Holbrook, AG 308

Holden, L 1849

Hole, DJ 505

Holford, TR 2087

Holick, CN 1705

Holland, CM 891

Hollaus, P 702

Holloway, S 653, 1650

Hollstein, M 687

Hölscher, AH 1508

Hombach, A 1130

Homma, J 1172

Honecker, F 2051, 2133

Honjo, Y 1971

Höopper, M 1766

Hop, WCJ 2045

Hopfner, R 120

Hopman, AHN 128

Hopper, JL 1661

Hori, K 1334

Horwich, A 36

Hoshial, H 831

Hoshino, H 1008

Hoskin, PJ 1290

Hosokawa, S 1545

Houf, M 2019

Houlston, RS 308, 1961

Houston, A 1345

Hoving, EW 128

Howard, D 1048

Hruban, RH 338

Hsiao, J-R 344

Hsieh, L-L 681

Hsu, CY 1320

Hsu, H-K 1202

Hsu, Y-J 146

Hu, SX 135

Hu, Y 1812

Hubalek, MM 1934

Huber, C 1855

Huddart, RA 1143

Hudelist, G 983

Hughes, JAI 860

Hull, WE 754

Humphreys, J 1961

Hunter, DJ 847

Hurks, MH 1914

Hutcheon, AW 966

Hutchinson, OC 1327

Hyršl, L 1067

Iaffaioli, RV 1013

Ianniello, GP 1013

Ichikawa, W 1486

Ikeda, T 557

Ikonen, T 1966

Iliopoulou, EG 1055

Illiano, A 1013

Imrie, KR 482

Innes, HE 43

Inokuchi, M 1750

Iredale, R 1834

Isaacs, SD 1524

Isaacs, WB 1524

Ito, H 922

Ito, K 803

Itoh, K 1079

Itoh, T 1042, 1736

Iwadate, Y 1896

Iwashima, A 803

Izumi, S 1709

Jacques, C 997

Jaehde, U 787

Jaffe, H 423, 502

Jager, MJ 1914

Jain, P 36

Jakab, F 465

James, JJ 284, 660, 1310

Jansen, B 1352

Janson, P-O 1298

Janssen, D 713

Janssen, JJWM 1162

Jansson, S 460

Jao, S-W 146

Jayson, GC 426, 1152

Jeanblanc, M 120

Jenkins, GJS 1314, 1729

Jenkins, V 1445

Jernström, H 482

Jeziorska, M 426

Jha, P 2078

Jiang, JD 1566

Jiang, S-Y 146

Jiang, Y 1743

Jiménez-Cervantes, C 2004

Jimeno, J 763

Jin, W 185

Jin, Y-T 344

Jirström, K 1920

Jöckel, K-H 1205

Johanson, V 1383

John, V 746

Johnston, C 487

Jones, DR 74

Jones, GDD 2271

Jones, HJ 398

Jones, PH 808

Jones, R 36

Jonker, JW 1776

Jorna, AS 357

Jost, C 2202

Kaaks, R 1697, 1814

Kadomatsu, K 1086

Käfer, G 2051

Kaffashian, F 1693

Kagamu, H 803

Kahán, Zs 465

Kaijser, M 1664

Kajiwara, Y 1802

Kaku, T 546

Kal, HB 2184

Kalden, JR 2234

Kalifa, C 2038

Kallio, JP 1266

Kalthoff, H 1714

Kamei, H 795

Kaminski, N 314

Kamm, YJL 754

Kaneko, K 152

Kang, MK 1473

Kang, S-W 907

Kanz, L 29, 2051

Kanzawa, T 922

Kao, E-L 1202

Kapischke, M 1714

Karagas, MR 952

Kariya, M 2293

Kasumi, F 1627

Katerinaki, E 1123

Kato, H 140

Kato, K 1042

Katoh, H 1042, 1736

Katsouyanni, K 1255

Kaur, K 1166

Kawabuchi, Y 1876

Kawaciuk, I 1067

Kawana, T 831

Kawarada, Y 1042

Kawaraya, M 1885

Kawasaki, T 730

Kaye, SB 1843

Kazama, S 1232

Ke, Y 672

Keane, K 2190

Keefe, KA 1062

Kelleher, DK 405, 2333

Kellokumpu-Lehtinen, P 1266

Kelly, JD 2312

Kelly, V 43

Kemp, H 2004

Kendall, CA 106

Kendall, GM 1215

Kendrew, J 1889

Kerr, DJ 944

Kerr, G 1837

Kessels, AGH 128

Khal, J 737

Khan, F 2197

Kheddoumi, N 178

Khedhaier, A 1502

Khokha, R 1817

Kieback, DG 1927

Kieffer, V 2038

Kiehlmann, PA 821

Kim, CJ 1958

Kim, DH 577

Kim, HS 1958

Kim, M-Y 907

Kim, SH 1958

Kim, SY 1958

Kim, TW 1865

Kim, WS 1865

Kindblom, L-G 460

Kinlen, LJ 1215

Kirby, JA 2312

Kitajima, T 1885

Kitching, R 1538

Kiura, K 795, 1885

Kjeldsen, K 1633

Klapper, W 713

Klein, B 997

Klein, M 641

Klijn, JGM 2289

Klingenstierna, H 460

Klironomos, G 2341

Kloft, C 787

Knight, LA 2299

Knoth, H 1405

Knoth, S 1405

Knox, WF 277

Knuth, A 997

Ko, YH 1958

Kobayashi, N 158

Kobayashi, T 1172

Kobayashi, Y 730

Koch, S 2133

Kochman, D 617

Kockelbergh, RC 2271

Kodani, T 1885

Koedoot, CG 2219

Köhne, C-H 997, 2051

Kohno, E 730

Koizumi, W 2207

Kok, TC 2045

Kölby, L 1383

Kollmannsberger, C 29, 1141

Komatsu, N 1079

Kondo, S 922, 1042, 1736

Kondo, Y 922

Konishi, K 152

Kooijman, SALM 2254

Korpela, J 971

Korst, AEC 2305

Kosmatopoulos, K 199

Köstler, WJ 983

Kotzerke, J 625

Koukourakis, MI 877

Koumenis, C 746

Kovalchuk, E 2098,

Koyama, K 1709

Kraemer, M 215

Kramar, A 1987

Kramer, H 1192

Krams, M 713

Kreklau, EL 1517

Krishna, NS 552

Kroon, BBR 243

Kropp, J 625

Kubat, B 128

Kubista, E 983

Küchler, Th 50

Kuh, D 852

Kujas, M 2327

Kulagenko, V 2098,

Kumar, S 426

Küpeliog̀lu, A 870

Kurisu, K 1802

Kurokawa, T 1042

Kusakari, T 2293

Kusenda, Z 617

Kutomi, G 1627

Kuwano, H 140

Kyo, S 922

Labbe, M 816

Labianca, R 1428

Labrinidis, A 206

Lacaze, E 2038

Laffer, U 422

Lafitte, J-J 55, 959

Lagiou, A 1255

Lagiou, P 1255

Lagrange, M 422

Lai, V 1428

Lajer, H 1633

Laking, G 224

Lambert, A 524

Lambert, PC 74

Lambin, P 1796

Lambrechts, HAJ 2305

Lampert, IA 113

Lancashire, RJ 1215

Landberg, G 1920

Landuyt, W 1796

Langlois, C 1638

Lapis, K 465

Larbouret, C 1987

Lardon, F 2305

Larsson, C 720

Larsson, O 720

Laskey, RA 1048

Latif, Z 1305

Lau, P 524

Lauretti, GR 2027

La Vecchia, C 1255

Lawlor, DA 81

Layton, C 2004

Lê, MG 840

Le Bouëdec, G 1185

Le Corre, L 168

le Monier, K 1096

Le TKP 363

Leaderer, B 2087

Lecluse, Y 577

Lecomte, T 1439

Lee, AHS 1310

Lee, H-P 1686

Lee, JH 1958

Lee, J-M 1202

Lee, JW 1958

Lee, JY 1958

Lee, KAW 1072

Lee, M-S 146

Lee, PWR 308

Lee, SH 1958

Lee, Y-C 320

Leede, GPJ 1192

Lehnert, M 1865

Lehrer, S 1815

Leitzmann, MF 1705

Lemaire, F 1940

Lemonnier, FA 199

Leng, G 2163

Leonard, P 2284

Leonard, RCF 1837, 2062

Leong, T 1433

Leporati, C 1457

Lessing, JB 109

Leuraud, P 2327

Levin, I 109

Levin-Jakobsen, A-M 1383

Lewis, AAM 308

Lewis, CE 421

Li, C 426

Li, CI 513

Li, H 524, 1538

Li, KKC 1072

Li, T 672

Liang, K 185

Liao, C-T 681

Liao, S-K 1072

Liepe, K 625

Lim, AG 308

Lim, Joon, D 1433

Lim, W 308

Lin, Y-C 1072

Lindelöf, B 1221

Lindholm, C 1517

Linn, S 1657

Little, MP 1215

Littlejohns, P 2163

Liu, B 185

Liu, VWS 697

Liu, XM 1566

Ljungberg, B 1285

Locher, C 1439

Loew, HG 937

Lofts, FJ 252

Lord, RV 1508

Lordick, F 630

Lorenzi, C 2038

Lorigan, PC 1123

Lorusso, D 1180

Lorusso, V 992

Louis, B 120

Love, SB 232, 431, 605, 781

Lovicu, FJ 860

Lowy, A 422

Lu, Y 185

Lu, Z-M 672

Lucas, T 1352

Lucassen, A 308

Ludicke, F 2023

Ludovisi, M 1180

Lui, W-O 720

Lundbeck, F 298

Luthra, SK 1327

Luukkaala, T 1266

Luwaga, A 65

Lynch, CF 1513

Lyratzopoulos, G 828

Ma, S 1517

Maaser, K 564

Mabro, M 1439

Macdonald, RC 308

MacGrogan, G 666

Mackintosh, D 1166

MacLean, AB 2249

MacMillan, C 1155

Macmillan, RD 1310

MacNeil, S 1123, 2004

Madersbacher, S 702

Madhavan, KK 1423

Madhusudan, S 1418

Madi, L 1552

Maeda, S 1614

Magagnoli, M 1159

Magdelénat, H 539

Magee, LRA 1022

Magné, N 585

Maione, P 1013, 1827

Maiorino, L 992

Maisey, N 36

Major, AL 2023

Makino, R 152

Makita, M 1627

Malkapuram, S 412

Malone, KE 513, 1513

Manavi, M 983

Mancarella, S 992

Mancuso, S 1180

Manda, R 140

Mandai, K 1802

Mandai, M 2293

Manders, P 271

Mangtani, P 508

Mann, JR 1200

Manning, M 930

Mant, D 2078

Manzione, L 1013

Maoleekoonpairoj, S 1865

Marchal, JA 192

Marchal, P 1940

Marchini, S 763, 1559

Marcucci, L 1091

Marcussen, N 298

Margalit, O 314

Mariadason, JM 1757

Marie, Y 2327

Mariggiò, MA 2244

Markham, C 1860

Marmot, MG 1243

Marples, M 1901

Marrocco, T 930

Marsh, HP 1096

Marshall, E 43

Marshall, SE 1096

Marsili, S 239

Marth, C 1934

Martikainen, PM 1266

Martin, A 215

Martin, B 55, 959

Martin, C 1581

Martin, LJ 1672

Martin, SJ 1327

Martineau, P 1987

Martínez, C 1667

Marubini, E 268

Maruyama, Y 803

Masaki, T 2104

Mascaux, C 55, 959

Massie, C 1837

Massuger, LFAG 886

Masuda, N 140

Mathoulin-Pélissier, S 666, 2213

Matikainen, MP 1966

Matsubara, S 1086

Matsuda, K 1232

Matsui, H 691

Matsumoto, K 831

Matsumoto, N 707, 2104

Matsumura, Y 816, 2207

Matsuura, S 1802

Mattern, J 2155

Mauriac, L 666

Mayer, á 465

Mayer, D 508

Mayer, F 2133

Mbidde, E 423, 502

Mbulaiteye, S 423, 502

McAdam, K 1155

McArdle, CS 1028

McArdle, PA 1906

McArdle, S 612

McCann, J 1693

McConkey, DJ 8

McCormack, VA 852

McCredie, MRE 1661

McDonald, A 1155

McEwan, C 333

McGlashan, ND 777

McGowan, R 1375

McGuckin, MA 524

McGurk, M 1610

McKee, KS 1366

McKelvey-Martin, VJ 2264

McKendrik, J 2190

Mckeown, A 277

McKeown, SR 2264, 2277

McMahon, L 1843

McMeekin, S 1062

McMillan, DC 612, 1028, 1906

McNally, OM 966

McNicol, A-M 1906

Mechinaud, F 470

Medioni, J 2327

Meert, A-P 55, 959

Mehring, G 1855

Meijer, CJLM 834, 1830

Meiler, S 2202

Meineke, V 2122

Meinhardt, P 1855

Meis-Kindblom, JM 460

Melchers, WJG 886

Melguizo, C 192

Melle, D 1940

Mellon, JK 2271

Melton, DW 333

Mendilaharsu, M 1209

Mercer, SJ 2299

Merino, P 1002

Merrick, AE 1276

Mertani, HC 524

Messinese, S 239

Mestiri, S 1502

Metzger, R 1508

Metzner, B 29

Mey, U 1413

Meyer, H 1352

Meyer, T 1901

Meyers, DA 1524

Meyskens, FL 1062

Michael, M 1433

Michaud, DS 1705

Michon, J 470, 1605

Micksche, M 702

Mignogna, MD 2244

Milano, G 585

Mild, G 422

Miles, D 2062

Miller, JL 937

Miller, MP 262

Millon, R 1940

Mills, GB 135

Milner, AE 944

Milosavljev, S 1855

Min, B-H 907

Minami, K 1876

Minkin, S 1672

Missitzis, I 1055

Mitamura, K 152

Mitry, E 1439

Miwa, K 676, 1750

Miyahara, E 1876

Miyake, M 158

Miyamoto, M 1042, 1736

Miyamoto, S 546

Miyao, H 803

Miyazaki, T 140

Mo, L 333

Mohammed, FF 1817

Mohn-Staudner, A 702

Molier, M 2289

Møller, H 492

Mõlne, J 2093

Monia, BP 1352

Monk, BJ 1062

Monnier, P 2320

Mononen, N 1966

Monté, D 120

Montembault, S 1439

Mooney, R 1979

Moore, MJ 617

Morales, J 1248

Morandini, R 2004

Mordenti, P 477

Morel, G 524

Morgan, C 1314

Mori, T 2104

Moriarty, M 954, 1979

Morimoto, C 1366

Morisaki, K 1971

Morizet, J 577

Morris, LS 1048

Mort, R 333

Morton, J 533

Morton, LM 2087

Mosconi, S 1428

Mosseri, V 1605, 2057

Mothersill, C 954, 1979, 2277

Mouret-Reynier, M-A 1185

Mousli, M 120

Mravunac, M 886

Mucci, LA 775

Mühlbauer, K-R 687

Muirhead, CR 1215

Mukherjee, P 1375

Muller, D 1940

Müller, HM 1934

Müller-Holzner, E 1934

Müller, R 983

Munakata, A 23

Muñoz, N 1209, 1667

Munzer, C 1605

Muramatsu, T 1086

Murday, V 308

Muro, K 816

Murray, PG 1200

Muth, A 2093

Muto, T 1232

Muzio, LL 2244

Nadjem, TA 1971

Nadolny, P 808

Nagaike, K 1545

Nagashima, F 2207

Nagashima, K 1072

Nagawa, H 1232

Nagayama, S 816

Nagley, P 697

Nagourney, RA 1789

Nakajima, M 23, 140

Nakakubo, Y 1736

Nakamura, H 730

Nakano, H 546

Nakashima, M 546

Nakata, S 691

Nakazato, H 691

Nam, SW 1958

Namba, H 1896

Nambooze, S 65

Nanbu, K 2293

Napier, MP 1901

Narita, M 1172

Nasiri, N 1035

Neale, K 308

Nehls, O 2051

Nelson, M 457

Neri, A 239

Neuzil, J 1822

Newell, DR 437

Newlands, ES 248, 1849

Newsom-Davis, T 457

Newton, R 423, 502

Ng, TY 697

Ngan, HYS 697

Ngan, S 1433

Nichelatti, M 465

Nicholas, C 1757

Nicklin, J 497

Nicola, S 1463

Niederle, N 997

Nielsen, HJ 420

Nierenberg, DW 952

Nieto, A 1667

Nigro, G 1091

Nihei, Z 1486

Niki, H 1545

Nilsson, A 1298

Nilsson, B 460

Nilsson, O 1383, 2093

Ninomiya, I 1750

Nishii, K 1885

Nishimura, S 1627

Nishiwaki, M 730

Niv, J 109

Noel, M 2069

Nohga, K 1545

Nojima, T 1072

Nord, C 1145

Norman, A 1143

Norman, AR 36

Nos, C 539

Noumaru, S 1614

Nozza, A 1159

Numata, Y 707

Nurmi, M 971

Nussey, F 2062

Nuyts, S 1796

O'Brien, SG 1855

O'Callaghan, A 2299

O'Connell, J 1345

Odajima, T 557

Oden, A 2093

O'Donohue, J 308

O'Dwyer, ST 426

Oechsle, K 2051

Oehler, MK 1927

Oestern, S 1714

Oetzel, C 2234

Ogi, K 557

Ohana, G 1552

Ohkubo, T 1008

Ohnuma, K 1366

Ohshita, A 1876

Ohta, K 1876

Ohtake, N 691

Ohtsu, A 2207

Ojeda, B 1002

Okasha, M 81

Okayasu, I 707

Okazawa, T 2116

Oki, A 831

Okimoto, N 795

Okita, K 2116

Okouoyo, S 2155

Okushiba, S 1042

Oleinick, NL 1590

Oliva, K 374

Oliveira, GM 2027

Oliver, RTD 1140, 1598

Olsen, JH 1513

Olshefski, RS 1743

O'Neill, GM 860

Opolon, P 199, 577, 1102

O'Reilly, SM 43

Oriana, S 268

Orive, G 1395

Orlowska-Volk, M 1927

Ortoncelli, M 1457

Osann, K 1062

Osella-Abate, S 1457

Oshikiri, T 1736

Osman, S 1327

Ossenkoppele, GJ 1162

Ostrander, EA 1530

Ostrowski, J 1493

O'Sullivan, GC 1345

Ota, H 152

Otake, M 730

Oudet, P 120

Out-Luiting, CJJ 1914

Owens, PH 2087

Oyen, R 1409

Oza, AM 617

Pääkkö, P 1270

Paci, E 1645

Paesmans, M 55, 959

Paganelli, G 930

Palmer, DH 944

Palmer, K 385

Palmeri, S 992

Palussière, J 2213

Pang, D 603

Pang, S-T 720

Pang, X-W 291

Pannone, G 2244

Pantaleo, MA 477

Papadaki, E 2341

Papamichail, M 1055

Papi, S 930

Paraskeva, C 1358

Park, I-S 907

Park, J 1508

Park, JY 1958

Park, M-O 907

Park, N-H 1473

Park, WS 1958

Parkes, S 1200

Parkin, DE 966

Parkin, DM 65, 423, 502

Parks, RW 1423

Parry, EM 1314, 1729

Parry, JM 1314, 1729

Parton, M 1035

Parvinen, L-M 971

Parwaresch, R 713

Pasquali, M 412

Patmore, HS 864

Pattyn, GGO 2305

Paul, J 1843

Paul, T 1620

Peake, MD 2190

Pedraz, JL 1395

Peintinger, F 648

Pèlegrin, A 1987

Pellegrini, M 199, 239

Pellizzaro, C 268

Penault-Llorca, F 1185

Peng, B 1855

Pereira, NL 2027

Perez, SA 1055

Perotti, C 173

Perret, G 215

Perrett, CW 2249

Perrone, F 1013

Perry, N 852

Peschel, C 630

Peters, GJ 754

Peters, J 915

Peterson, J 1255

Peto, J 2078

Petrinelli, P 1091

Petrioli, R 239

Petty, RD 966

Pfeffer, R 314

Philip, R 222

Philips, R 1155

Phillips, RKS 308

Piantedosi, FV 1013

Piazza, E 1013

Picot, V 666

Pierga, J-Y 539, 2057

Pike, M 2078

Pinder, SE 284, 660, 1310

Pinedo, HM 1162

Pinto, E 239

Piselli, P 94

Pisvin, S 899

Plantaz, D 1605

Polee, MB 2045

Polesel, J 94

Popowski, GY 2023

Porteous, M 653, 1400, 1650

Porter, D 615

Porter, PL 513

Portin, R 971

Poruchynsky, MS 1971

Potter, CPS 2

Pouillart, P 539

Poupon, M-F 2327

Powell, JE 1200

Powles, T 457, 1140

Pozzessere, D 239

Prados, J 192

Prasad, R 277

Preusser, P 997

Price, ME 2264, 2277

Price, P 262, 1327

Pringle, J 2284

Pritchard-Jones, K 327

Procaccini, M 2244

Propper, D 262, 1418

Protheroe, A 1418

Puchkou, A 2098,

Punt, CJA 754

Purdue, MP 951

Purohit, OP 2031

Qian, W 713

Qian, X-P 291

Qiao, H-Z 672

Quaglino, P 1457

Quagliuolo, V 1159

Quietzsch, D 2051

Quinn, M 374, 508

Quint, WGV 886

Quintana, MJ 1667

Rabelink, MJWE 2140

Raccurt, M 524

Radecki, S 1789

Radny, P 1620

Radu, A 2320

Radzun, HJ 915

Ralph, G 648

Ramaekers, FCS 128

Ramanakumar, AV 65

Ramos, JL 192

Ramsamooj, R 2110

Rask, K 1298

Ratanatharathorn, V 1865

Rath-Wolfson, L 1552

Raulf, F 2019

Ravaud, A 2213

Rea, D 1155

Rechavi, G 314

Redfern, D 1200

Redhead, DN 1423

Reece, WHH 1865

Reed, N 957, 1843

Rees, MCP 1927

Reeves, G 423

Reid, WMN 2249

Reiman, A 1200

Reitsamer, R 648

Reitz, M 50

Remadi, S 1502

Renée, N 585

Renehan, AG 1610

Repp, R 2234

Reshkin, SJ 1395

Reuter, J 422

Reversi, A 930

Rezza, G 94

Rice, G 374

Richards, FJ 1155

Rick, O 29

Ringberg, A 1920

Ringborg, U 1517

Risbood, M 1971

Ritchie, AWS 1598

Riva, A 1463

Rizvi, I 937

Robaye, B 727

Robbiati, SF 1013

Robert, B 1987

Robert, J 666

Robertson, JFR 284, 660

Robey, RW 1971

Robinson, A 1155

Robinson, D 492

Robinson, MH 308

Robinson, S 1389

Robson, L 1152

Robson, T 2264, 2277

Rochlitz, C 422

Röder, C 1714

Rodriguez, AC 1248

Rofstad, EK 350

Rökman, A 1966

Rolfe, KJ 2249

Roman, E 1228

Ronco, AL 1209

Roozendaal, KJ 243

Rosa, GD 2244

Rose, GS 1062

Rosenstein, BS 1513

Rosetti, F 1013

Rosmorduc, O 252

Ross, JA 602

Rosselli, Del, Turco, M 1645

Rossi, A 1827

Rothnie, A 1581

Rotilio, D 763

Rozendaal, L 834, 1830

Rubie, H 470, 1605

Rubini, C 2244

Rubino, C 840, 1638

Rudolph, P 666

Runge, R 625

Runge, S 1971

Rush, R 653, 1650

Rushbrook, SM 1048

Russo, G 1091

Rustin, GJS 808, 1849, 1901

Rydh, B 1221

Sabatino, M 239

Sacks, N 1035

Saeed, IT 1048

Sahin, F 338

Saigenji, K 2207

Saijo, N 2178

Saiki, Y 2116

Sainsbury, R 15, 487

Saisho, H 1086

Saito, H 23

Saito, S 1334

Sakiyama, S 1086

Salminen, E 971

Salmon, RJ 2057

Salomon, Y 2333

Salonga, D 1508

Salter, J 1035

Samoli, E 1255

Samouri, K 1166

Sánchez, MJ 1667

Sánchez-Carbayo, M 2172

Sanderson, TM 1375

Sandison, A 2284

Sanson, M 2327

Santegoets, SJAM 1162

Santini, D 1428

Santoro, A 1159

Sanyal, U 2165

Sápy, P 465

Sarid, R 1657

Sarina, B 1159

Sariogǧlu, S 870

Sasaki, Y 1486

Sasieni, P 88

Sato, K 1366, 1614

Sato, N 338

Sato, T 803

Satoh, M 557

Satoh, S 158

Sauerbruch, T 1413

Saunders, MP 426, 1155

Savoia, P 1457

Sawamizu, H 1079

Sawamura, A 1876

Scambia, G 1180

Scardino, A 199

Schaefer, K-L 1072

Schalkwijk, C 357

Schatzl, G 702

Scheper, RJ 1162

Scherübl, H 564, 1766

Scherz, A 2333

Schiffman, M 1248

Schijf, CPT 886

Schittenhelm, M 2133

Schlegel, C 1620

Schleiermacher, G 470

Schleucher, N 29

Schleutker, J 1966

Schlie, C 1413

Schlieman, M 391

Schlieman, MG 2110

Schliep, S 2147

Schlumberger, M 1638

Schmidt-Wolf, IGH 1413

Schmiedel, S 1714

Schmoll, H-J 29

Schnattinger, K 702

Schneider, PM 1508

Schroth, A 2155

Schultz, I 1940

Schulz, D 1405

Schutte, M 2289

Schvartz, C 1638

Schwartz, GG 746

Schwarz, M 1620

Schweyer, S 915

Sciot, R 1409

Scott, IS 1048

Scuffham, P 634

Sculier, J-P 55, 959

Scully, J 15

Searle, KM 437

Secher, NH 1633

Seckl, MJ 1849

Segawa, Y 795

Seiffert, B 2147

Sekine, K 1008

Selzer, E 1352

Seppälä, EH 1966

Serafini, M 763

Seth, A 1538

Seyfried, TN 1375

Seymour, CB 954, 1979

Seymour, MT 1155

Shah, B 308

Shah, P 457

Shah, V 1729

Shamash, J 1140

Shamsaldin, A 840

Shanahan, F 1345

Shani, A 997

Sharp, P 224

Sharp, T 2284

Sharples, LD 1022

Sheen, J-H 1479

Sherk, AB 746

Sherlock, D 426

Sherman, ME 1248

Sherwood, BT 2271

Shiau, A-L 344

Shibata, J 1614

Shibayama, T 1885

Shichijo, S 1079

Shields, TS 1248

Shih, J-Y 320

Shih, Y-L 146

Shimada, Y 816

Shimamura, T 152

Shimizu, K 1876

Shin, K-H 1473

Shin, Y-J 907

Shinohara, T 1042

Shinozaki, M 1232

Shipley, J 327

Shipley, MJ 1243

Shirao, K 816, 2207

Shirota, Y 1486

Shoenfeld, Y 465

Shohat, M 314

Short, D 1849

Shyu, R-Y 146

Sibtain, A 1290

Siddiqui, N 957

Sidell, N 412

Sidorov, Y 2098,

Siegert, W 787

Sierankowski, G 666

Siersema, PD 2045

Silberman, D 1552

Silbermann, M 1657

Simoens, C 2305

Simon, R 1599

Simpson, J 1228

Singer, CF 983

Singh, AD 1914

Siracusano, L 1159

Siu, LL 617

Sivridis, E 877

Skov, T 252

Skovsgaard, T 252

Slosman, G 1552

Smartt, HJM 1358

Smith, DB 43

Smith, HJ 1783

Smith, IE 1035

Smith, JS 101

Smith, KC 1530

Smith, LK 70, 74

Smith, SK 891

Sobue, T 23

Soda, M 1709

Sohda, M 140

Sökmen, S 870

Solis-Trapala, I 1445

Solomon, L 1693

Soma, Y 23

Sombroek, CC 1162

Sommers, BL 1789

Sonoda, K 546

Sorahan, T 1215

Soruri, A 915

Sotiropoulou, PA 1055

Soto, Parra, H 1159

Soukop, M 1152

Soung, YH 1958

Southey, MC 1661

Span, PN 271

Spector, LG 602

Spencer, E 519

Sperduti, I 1870

Spix, C 1260

Splinter, TAW 2045

Springer, ING 1722

Stafford, ND 864

Staibano, S 2244

Stalmeier, PFM 2219

Stam, AGM 1162

Stang, A 1205

Starzec, A 215

Stattin, P 1814

Stearns, ME 1812

Steel, M 653, 1650

Steels, E 55

Stefanou, D 2341

Stefoski, Mikeljevic, J 487

Steger, GG 983

Stegmaier, C 1205

Steineck, G 775

Steininger, H 2234

Stevenson, L 821

Steward, WP 70, 252, 2271

Stewart, J 1155

Stierner, U 460, 1517

Stiggelbout, AM 2219

Stocken, DD 1860

Stockmeyer, B 2234

Stone, J 1672

Stone, N 106

Stoodley, BJ 308

Stoter, G 2045

Stranzl, H 648

Straume, O 1031

Strauss, SJ 1901

Strehl, J 1413

Streri, A 2038

Strijbos, JH 1192

Sturla, L-M 1276

Su, GH 338

Su, W-C 344

Su, Y-R 291

Subramaniam, V 1538

Sugihara, K 1486

Sugiyama, M 2104

Suhr, MAA 1722

Sullivan, DC 228

Sun, C-L 1686

Sundfeldt, K 1298

Sutter, AP 564, 1766

Suyama, A 1709

Suzuki, E 803

Suzuki, K 691, 1232

Suzuki, Y 2104

Suzuoki, M 1736

Swarbrick, ET 308

Sweep, CGJ 271

Sweetland, S 519, 2078

Swerdlow, AJ 852

Symonds, RP 2271

Szabo, M 2004

Szentpétery, F 465

Tabata, M 795, 1885

Tada, K 1627

Tagaki, S 1008

Tagawa, M 1086

Tagawa, T 1545

Taillandier, L 2327

Takabatake, H 1008

Takabayashi, A 158

Takahashi, M 1172

Takahashi, T 1486

Takakura, K 2293

Takedatsu, H 1079

Takemoto, M 795

Takeo, S 2116

Taki, T 158

Takigawa, N 795

Talaban, R 1961

Talbot, DC 808, 1166

Talbot, I 308

Tallini, G 2087

Talvensaari-Mattila, A 1270

Tam, SP 524

Tamaoki, T 1086

Tammela, TLJ 1266, 1966

Tams, C 1714

Tanabe, M 1627

Tanabe, S 2207

Tanaka, J 803

Tanaka, M 23, 1614

Tanaka, N 557

Tanaka, R 1172

Tang-Liu, D 808

Tanimoto, M 795, 1885

Tanioka, Y 2116

Tannock, IF 641

Tattersall, M 2069

Tauchi, H 1802

Taylor, M 1166

Tchen, N 641

Tebeu, PM 2023

Teitelbaum, SL 1513

Telekes, A 465

ten Velden, JJA 243

Terada, I 1750

Terheyden, H 1722

Terracciano, L 422

Terzoli, E 1870

Tessier, JJ 1889

Testa, A 1180

Testa, E 1428

Thallinger, C 1352

Thatcher, N 2190

the Danish RANX05 Corolectal Cancer Study Group, 420

The Uganda Kaposi's Sarcoma Study Group, 423, 502

Thews, O 405, 2333

Theys, J 1796

Thiery, J-P 539

Thirwell, C 457

Thomas, BL 1610

Thomas, C 1605

Thomas, G 1155

Thomas, M 29

Thomas, R 653, 1433, 1530, 2062

Thomasson, M 1285

Thompson, A 1433

Thompson, P 615

Thompson, WD 1513

Thomson, BNJ 1423

Thomson, CS 2031

Threlfall, AG 77

Thurzó, L 465

Thuss-Patience, P 997

Tiemann, M 1722

Tiffin, N 327

Tiffon, C 585

Tilanus, HW 2045

Tillman, BW 1162

Timofeeva, I 1159

Tipton, KF 1979

Tirmarche, M 2098,

Tisdale, MJ 737, 1116, 1783

Tisell, LE 2093

Toge, T 1876

Tognon, G 763

Tokudome, N 1627

Tokuhara, T 158

Tomita, K 1614

Tomizawa, M 1086

Tomlinson, I 308

Tompkins, K 482

Tonini, G 1428

Törnqvist, M 774

Touzé, A 423

Trauzold, A 1714

Tretli, S 1238

Trichopoulos, D 1255

Trieb, K 702

Trufflandier, N 2213

Tsai, S-T 344

Tsang, PCK 697

Tschada, R 687

Tsuchiya, N 1172

Tsugawa, K 1750

Tsukada, K 140

Tsuruta, Y 2293

Turner, A 1418

Turner, AR 1524

Turpeenniemi-Hujanen, T 1270

Tursi, J 1559

Twelves, C 1155

Twijnstra, A 128

Ucur, E 2155

Ueno, T 1517

Ueoka, H 795, 1885

Uetake, H 1486

Uff, JS 106

Ummelen, MIJ 128

Underwood, MA 1305

Ungefroren, H 1714

Usel, M 2023

Usuda, J 1590

Vaccari, M 1463

Vágvölgyi, A 465

Vainio, H 1697

Valentine, H 870

Valenty, M 2098,

Valerius, T 2234

Vallot, F 55

Valteau-Couanet, D 1605

van Ballegooijen, M 1830

van Beuningen, D 2122

van Beusechem, VW 577

van Bezu, J 357

van de Loosdrecht, AA 1162

van den Akker-van Marie, ME 1830

van den Bergh, H 2320

van der Burg, MEL 2045

van der Gaast, A 2045

van der Valk, MA 1776

van der Velden, PA 1914

van der Vijgh, WJF 357, 2140

van der Woude, HJ 243

van de Winkel, JGJ 2234

van Geelen, CMM 363

Van Hecke, P 1796

van Leeuwen, IMM 2254

van Nieuwpoort, FA 1914

van Ojik, HH 2234

Van Oosterom, A 1409

van Putten, JWG 1192

van Tellingen, O 1776

van Weeghel RP 363

Vanhentenrijk, V 1409

Vanni, B 1870

Varakis, J 2341

Vasey, P 1152, 1418

Vasey, PA 1843

Vassal, G 577, 1108

Vassy, R 215

Vaughan Jr, ED 1598

Vaupel, P 405, 2333

Vayre, L 1439

Veélez, C 192

Veltri, E 1013

Velu, T 727

Veneroni, S 268

Venter, DJ 1661

Vercelli, M 94

Verdebout, J-M 55, 959

Verheijen, RHM 834

Verheul, HMW 357

Verkooijen, HM 2023

Vermorken, JB 2305

Vernimmen, D 899

Vernon, DI 398

Vessey, MP 2078

Villa, ML 1463

Vincent-Salomon, A 539

Vinnicombe, S 852

Virudachalam, S 391

Viscidi, RP 1248

Visioli, C 1645

Vissac-Sabatier, C 168

Visser, O 834

Voelter, V 1055

Vogt, KN 1672

Vollmer, TC 2202

von der Maase, H 298

von Rosen, A 720

von Schilling, C 630

Vowler, SL 1048

Vries, EGE 1192

Vutuc, C 702

Vyas, S 308

Wabinga, H 65, 423, 502

Wacheck, V 1352

Wachters, FM 1192

Wada, A 1086

Wada, RK 412

Wadd, NJ 1155

Wagner, A 915

Wagner, M 2004

Wagnières, G 2320

Walker, J 1581

Walker, P 808

Walsh, PC 1524

Wandert, T 50

Wang, C-H 1072

Wang, JJ 1995

Wang, JP 135

Wang, LG 1566

Wang, M 1812

Wang, S 135

Wang, SS 1248

Wang, Y 374, 697, 1865

Wang, Z-B 2227

Wängberg, B 460, 1383

Wanichkul, T 412

Ward, S 634

Warnke, P 1722

Waroquier, D 727

Warren, E 634

Wasylyk, B 1940

Wasylyk, C 1940

Watanabe, K 1086

Watanabe, T 546, 1232, 2104

Watanabe, Y 795

Waters, MJ 524

Waterton, JC 1889

Watters, AD 1305

Waxman, J 779

Wedge, SR 1889

Weide, B 1620

Weiderpass, E 1697

Weih, L 1433

Wein, A 2051

Weinberger, RP 860

Weirich, G 2147

Weiss, R 502

Weiss, RA 223, 425

Wells, AJ 955

Wells, P 262

Welsh, KI 1096

Wen, B 1102

Weng, W-H 720

Wenger, T 2155

Wersinger, EM 968

Werther, K 420

Wessels, PH 128

West, C 870

West, CR 828

West, M 15

Westers, TM 1162

Westphal, S 1714

Westwood, NB 437

Weyandt, GH 2019

Wheatley, DN 222, 573, 907

Whelan, J 2284

Whitehouse, AS 737, 1116

Whitehouse, PA 2299

Widschwendter, A 1934

Widschwendter, M 1934

Wieland, G 2234

Wiersma-van, Tilburg, A 886

Wigmore, SJ 1423

Wilczynski, SP 1062

Wiley, KE 1524

Wilke, HJ 997

Willett, WC 847, 1705

Williams, BG 1834

Williams, EMI 828

Williams, NA 1358

Williams, RD 327

Wilson, AJ 1757

Wilson, ARM 1310

Wilson, GD 1290

Winkler, R 899

Winsey, SL 1096

Winstone, K 497

Wixey, J 1961

Wojcicki, JM 2016

Wong, LC 697

Wong, M 1538

Wong, N 1031

Woo, J-K 1479

Woodman, CBJ 77

Workman, P 1327

Wort, R 1961

Wotherspoon, A 36

Wouters, B 1796

Wrana, J 1538

Wright, MPJ 106

Wright, T 1248

Wu, C-L 344

Wu, C-T 320

Wu, D-C 1202

Wu, F 2227

Wu, H-Y 291

Wu, L 185

Wu, M-T 1202

Wu, Q-J 672

Wyld, L 284

Xu, H-J 135

Xu, J 1524

Xue, L-Y 1590

Xue, SA 113

Yagawa, T 2116

Yager, ML 860

Yajima, N 1172

Yamada, H 1486

Yamada, Y 816

Yamaguchi, Y 1876

Yamamoto, T 152

Yamanaka, H 691

Yamanaka, R 1172

Yamaoka, Y 158

Yamasaki, F 1802

Yamashita, K 707

Yamaura, A 1896

Yamochi, T 152, 1366, 1366

Yanai, H 2116

Yang, HJ 697

Yang, W 1757

Yang, X-A 291

Yap, JT 262

Yasugi, T 831

Ychou, M 1987

Yi, Q-L 617, 641

Yokoyama, T 1232

Yoo, NJ 1958

Yoshida, T 707, 2116

Yoshikawa, H 831

Yoshimoto, M 1627

Yoshioka, H 1802

Yoshizawa, H 803

Young, DA 1812

Young, ID 308

Young, J 1940

Young, LS 944, 1200

Youssef, M 1062

Yu, J-C 146

Yu, L 1086

Yu, MC 1686

Yu, Z 808

Yuan, J-M 1686

Yukelson, A 1657

Yung, BYM 1320

Zahm, SH 2087

Zaknoen, S 248

Zalcberg, J 1433

Zanetti, R 94

Zantl, N 2147

Zappa, M 1645

Zappalà, AMR 1870

Zat'ovičová, M 1067

Závada, J 1067

Závadová, Z 1067

Zeciri, G 2023

Zeitz, M 1766

Zhang, B 2087

Zhang, Y 2087

Zhao, C 1940

Zheng, SL 1524

Zheng, T 2087

Zhou, Y 135

Zhu, H 2227

Zidek, T 702

Ziegler, J 423, 502

Zielinski, CC 983

Ziske, C 1413

Zonneveld, C 2254

Zou, J-Z 2227

Zubovits, J 1538

Zuidervaart, W 1914

Zupi, G 1091

Zurbriggen, R 199

